# QproMS: a web application for label-free proteomic data analysis

**DOI:** 10.1093/bioadv/vbag158

**Published:** 2026-06-05

**Authors:** Fabio Bedin, Giorgia Cucina, Giampaolo Martinello, Stefano Rizzieri, Andrea Graziadei, Alessandro Cuomo

**Affiliations:** Department of Experimental Oncology at IEO, European Institute of Oncology IRCCS, Milan 20139, Italy; Department of Experimental Oncology at IEO, European Institute of Oncology IRCCS, Milan 20139, Italy; Department of Experimental Oncology at IEO, European Institute of Oncology IRCCS, Milan 20139, Italy; Department of Experimental Oncology at IEO, European Institute of Oncology IRCCS, Milan 20139, Italy; Human Technopole, Milan 20157, Italy; Department of Experimental Oncology at IEO, European Institute of Oncology IRCCS, Milan 20139, Italy

## Abstract

**Motivation:**

Proteomics has experienced substantial growth in methods and data analysis approaches, with the development of new data-dependent (DDA) and data-independent acquisition (DIA) workflow and several search engine algorithms and software packages. Each of these workflows has its unique data analysis package that performs data reduction, missing value imputation, statistical testing, and visualization. Often, these tools are designed for expert users.

**Results:**

We present Quantitative Proteomics Made Simple (QProMS), a user-friendly, search engine-agnostic data analysis and visualization pipeline. QProMS guides the user through data analysis and statistical testing in a graphical interface. Statistical tests rely on established R functions and are compatible with all types of label-free quantification experiments. The pipeline recapitulates features from different available software packages and introduces mixed imputation, an improved framework for handling missing values that does not rely on machine learning. QProMS can also perform interaction analyses based on gene ontology, or by querying protein-protein interaction databases. All figures in QProMS are interactive, allowing for investigation of individual proteins of interest before export. The analysis can be saved in a standalone report. QProMS provides a platform for reproducible proteomic data analysis for novice and experienced users, enabling state-of-the-art data analysis of a wide variety of label-free proteomic workflows ranging from global proteome profiling to targeted methods such as proximity labeling.

**Availability and implementation:**

QProMS is accessible as a web server hosted at https://shiny.bioserver.ieo.it/app/qproms or can be run locally as a standalone Shiny application with the code and instructions provided at https://github.com/ieoresearch/QProMS. The application may also be run locally by installing it as a library/package and running a single command as described in the README. Code to generate benchmarking is available at https://github.com/grandrea/mixed-imputation-benchmark.

## 1 Introduction

Label-free mass spectrometry (MS)-based proteomic workflows have become a central tool in the characterization of biological systems. Both data-dependent acquisition (DDA) and data-independent acquisition (DIA) strategies are now routinely used in combination with a wide range of proteomic search engines, enabling the identification and quantification of thousands of proteins from limited amounts of biological material. While each search engine carries out quantification in a slightly different manner, a common set of analyses is performed post-search to perform sample quality control, data filtering, and characterization of the underlying biology of the sample.

Current tools to perform such analyses tend to be tailored to individual search engines or specific workflows. Several tools are available for downstream analysis of label-free proteomics data, including Perseus (Tyanova *et al.* 2016b), LFQAnalyst (Shah *et al.* 2020), FragpipeAnalyst (Hsiao *et al.* 2024), and AlphaPeptStats (Krismer *et al.* 2023), as well as workflow-specific pipelines embedded in individual search engines. These approaches typically provide core functionalities such as data filtering, missing value imputation, statistical testing, and visualization for differential protein abundance analysis. However, they are often restricted to specific software ecosystems, quantification strategies, or acquisition modes, and may require the use of separate tools for functional enrichment, network analysis, or the comparison of complex experimental designs. For example, Perseus is implemented as a closed-source Windows application and is tightly coupled to the output of MaxQuant (Cox and Mann 2008). While users can save analysis sessions in a custom format, Perseus does not generate shareable reports that capture all steps required to fully reproduce an analysis. At the other end of the spectrum, FragPipeAnalyst provides a clean, modern graphical interface but requires an experimental design file formatted according to FragPipe (Kong *et al.* 2017) standards and lacks advanced functionality for functional enrichment and network analysis, necessitating the use of external tools.

**Figure 1:**
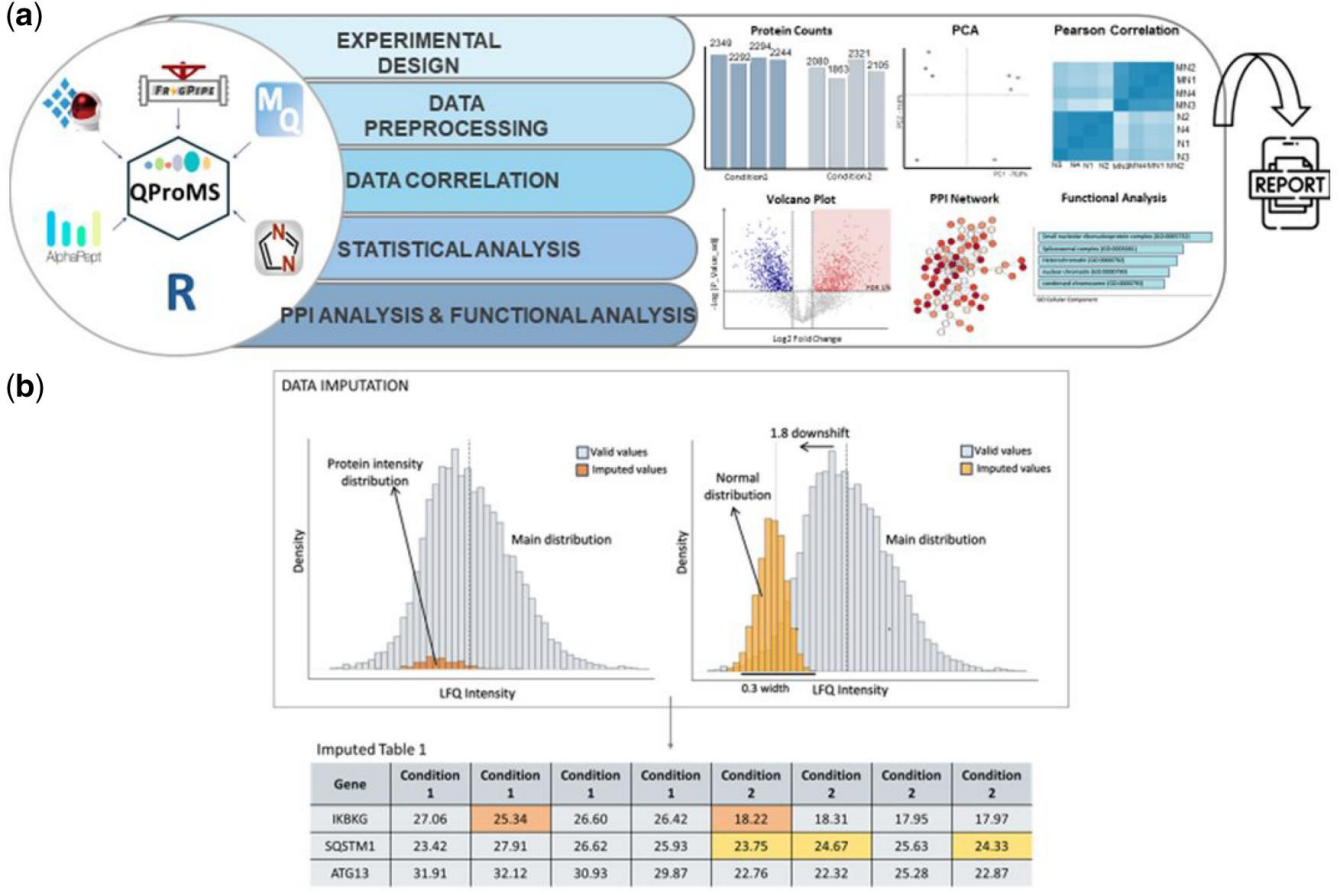
Overview of the QProMS workflow and mixed imputation strategy. (a) The Quantitative Proteomics Made Simple (QProMS) platform provides an end-to-end solution for the analysis of label-free proteomics datasets, supporting multiple search engine outputs (e.g. MaxQuant, FragPipe, AlphaPept, Spectronaut, DIA-NN). The pipeline comprises five key modules: experimental design, data preprocessing, data correlation, statistical analysis, and functional/protein–protein interaction (PPI) analysis. (b) The mixed imputation approach. Left, traditional (Perseus-like) downshift imputation applied uniformly to missing values; right, QProMS’s default mixed imputation approach, which classifies missing values as Missing At Random (MAR) or Missing Not At Random (MNAR) and imputes accordingly, MARs using a uniform distribution and MNARs using a downshifted Gaussian distribution. A sample imputed intensity table is shown for selected proteins across two conditions.

We present a fully open-source, cross-search engine compatible analysis pipeline for label-free proteomic workflows, Quantitative Proteomics Made Simple QProMS. QProMS is implemented in a ShinyApp web framework available at https://bioserver.ieo.it/shiny/app/qproms. The app may also be run locally with instructions, documentation and code available at https://github.com/ieoresearch/QProMS. Presented with protein abundance tables from MaxQuant (Tyanova *et al.* 2016a), FragPipe (Kong *et al.* 2017), AlphaPept (Strauss et al. 2024), Spectronaut or Dia-NN (Demichev *et al.* 2020), ProteomeDiscoverer (Orsburn 2021), the app guides the user through data reduction, imputation, and statistical analysis (correlation analysis, principal component analysis, ANOVA, *t*-test, multivariate analysis and enrichment analysis) (Fig. 1a). Additionally, the user may provide any table in custom format and manually select gene ID and intensity columns, allowing the app to support any search engine format. All statistical tests are based on established R functions, in a way similar to that of AlphaPeptStats (Krismer *et al.* 2023).

The app also provides new functions, such as the implementation of variance-stabilizing normalization (VSN), which has been shown to be the best method to normalize abundance across samples in DDA-based proteomics (Huber *et al.* 2002, Välikangas *et al.* 2018). Similarly, since DDA suffers from a high number of missing values due to the partially stochastic nature of precursor selection, we introduce a new method, mixed imputation, that distinguishes between missing at random (MAR) and missing not at random (MNAR) in the imputation procedure (Jin *et al.* 2021).

In addition to quality control and statistical analysis, QProMS can also perform functional analysis, including gene ontology and network analyses. This is handled by connecting to databases via OmniPath (Türei *et al.* 2021), allowing for the extraction of both functional (Gene Ontology, KEGG, WikiPathways) and physical interaction networks from STRING and CORUM (Szklarczyk *et al.* 2019, Tsitsiridis *et al.* 2023). These are particularly critical in enrichment experiments such as affinity purification-MS (Dunham *et al.* 2012) or proximity labeling (Qin *et al.* 2021), where the user is seeking to characterize the interactome of a particular target protein, making QProMS uniquely suited to analyzing these types of experiments. The networks displayed in QProMS are interoperable and can be exported for further analysis in CytoScape (Shannon *et al.* 2003). As QProMS is based on the plotting backend echarts4r, all analyses and visualizations in QProMS are interactive, allowing communication between the data tables and the plots.

In implementing an application that adheres to the FAIR principles for research software (Barker *et al.* 2022), we sought to make the whole analysis workflow fully reproducible by allowing the user to export both a report and the set of all parameters used in the analysis for re-uploading later. This also enables the user to save standardized workflows. Finally, the back end of the app is also implemented as a virtual environment in an R6 class that allows for the establishment of automated offline pipelines.

## 2 QProMS workflow

### 2.1 Data upload and missing values analysis

QProMS is implemented as a web application. The user is met with an upload page where the protein abundance file coming out of the proteomic search engine is uploaded and the experimental design is defined. QProMS natively handles MaxQuant (proteinGroups.txt), FragPipe (combined_protein.tsv), AlphaPept (results_proteins.csv), Dia-NN (unique_genes_matrix.txt), and Spectronaut (Report.tsv) outputs. Alternatively, the user can upload a custom table of gene names and protein abundances. For each of these programs, all label-free protein quantification methods can be selected as a basis for QProMS analysis (Label Free Quantification, iBAQ; Schwanhäusser *et al.* 2011), MaxLFQ (Cox *et al.* 2014), Intensity, MS2-based quantification, QuantUMS (Kistner et al. 2023). The experimental design is then handled interactively, with no additional uploads required.

After data upload, the user is presented with an analysis of data completeness and missing values across replicates and conditions based on distributions and UpSet plots. Here, the user can optionally choose to normalize abundances using variance-stabilizing normalization (VSN) (Huber *et al.* 2002, Välikangas *et al.* 2018). Additionally, data can be filtered based on criteria such as minimum number of peptides observed.

### 2.2 Imputation

Proteomics is especially affected by missing values, especially in DDA workflows (Karpievitch *et al.* 2009, 2012). In order to perform enrichment analysis, missing values are often imputed by a variety of approaches. One of the most popular approaches involves modeling the log-transformed intensity distribution of the sample as a normal distribution and imputing missing values by sampling from a down-shifted normal distribution (DSRD) to model low-abundant proteins. This imputation strategy is implemented in Perseus (Tyanova et al. 2016b). While straightforward and robust, this method has been shown to introduce a high normalized root mean squared error, leading to a drop of imputation accuracy and a high number of false positive identifications when performing enrichment analysis (Jin *et al.* 2021, Harris *et al.* 2023). The weakness of this method is that MNAR and MAR values are treated in the same way, when in reality they arise from different mechanisms. MNARs are more likely to be due to a protein abundance below the detection limit of the experiment, while MARs can arise due to the stochastic nature of DDA precursor selection and imperfect matching between runs in algorithmic searching for example (Lazar *et al*. 2016).

The approach introduced in QProMS, mixed imputation (Fig. 1b), assigns each missing value as MAR or MNAR based on the consistent presence or absence of the value among replicates. If a value is randomly missing (present in >75% of replicates within a condition), it is imputed by sampling from a uniform distribution between the minimum and maximum abundance within that condition. For a value present in <75% of replicates, the common down-shifted distribution sampling method is applied instead. The threshold of present values of 75% for differentiating MAR and MNAR in the mixed imputation is also exposed as a parameter that the user may adjust.

The app still leaves the user flexibility in choosing imputation strategy, allowing a choice of mixed imputation, DSRD, or a non-parametric imputation strategy based on a random forest implemented in the package missForest (Stekhoven and Bühlmann 2012). MissForest was shown to outperform DSRD in terms of root mean squared error and recall in differential analysis (Jin *et al.* 2021, Harris *et al.* 2023). The relative performance of DSRD imputation, mixed imputation and the more computationally expensive missForest is shown in Fig. 1, available as supplementary data at *Bioinformatics Advances* online. We tested a proximity labeling enrichment experiment (PRIDE identifier PXD043638) (Russo *et al.* 2023), and a global proteomics experiment of a 3-proteome mix used in previous benchmarking (Jin *et al.* 2021). Mixed imputation reliably outperforms DSRD imputation and approaches the more computationally expensive missForest in this limited test. Both dataframes are included as Supplementary Data 1, available as supplementary data at *Bioinformatics Advances* online. The benchmark shows mixed imputation is particularly suited to experimental designs like affinity purification-MS (AP-MS) and proximity labeling, where missingness is unequally distributed among conditions.

### 2.3 Statistical testing

The backbone of QProMS is a suite of statistical tests to perform the various analyses and visualizations required by proteomic workflows. Analysis of variance (ANOVA), multivariate analysis, principal component analysis (PCA), and univariate analysis with *t*-tests are implemented using standard R functions. The settings are displayed in a beginner-friendly panel, with documentation supporting the choice of test accessible on the GitHub repository. The application automatically produces volcano plots, heatmaps, profile plots, network analysis and geneset enrichment and over-representation analysis.

For univariate enrichment analysis, QProMS implements the simultaneous visualization of multiple volcano plots, which is particularly helpful in time series experiments or experiments probing multiple conditions or controls. Correction of *P* values for multiple testing can be performed with a number of strategies, such as Benjamini & Hochberg FDR (Benjamini and Hochberg 1995). Besides this, we implemented moderated *t*-testing with an empirical Bayes test, limma, that has been shown to achieve high sensitivity in two-group comparisons (Smyth 2004, van Ooijen *et al.* 2018).

Multivariate analysis and clustering by condition and/or protein is also implemented. The results are displayed in heat maps and profile plots to allow the user to identify clusters of co-regulating proteins.

### 2.4 Functional and network analysis

A strength of QProMS is the native integration of database-based information with statistics from quantitative proteomics via OmniPath (Türei *et al.* 2016). All lists from statistical analyses (up-regulated proteins, down-regulated proteins, all proteins, topN most abundant etc.) can be selected as the basis for Gene Ontology (GO) enrichment analysis, pathway analysis or interactome analysis. Human readability of GO enrichment is enhanced by allowing the user to select the level of aggregation of terms using the package clusterProfiler (Wu *et al.* 2021). The enriched terms in either over-representation analysis or geneset enrichment analysis are then visualized in bar plots that allow for comparison of multiple conditions. In a similar fashion, the user may select the KEGG database (Kanehisa *et al.* 2025) or WikiPathways (Miller *et al.* 2022) as sources for pathway enrichment analysis.

In addition to GO terms and KEGG/WikiPathways, protein-protein interactions in each list coming out of statistical analysis may be visualized based on STRING pairwise functional interactions and CORUM complexes, allowing the user to retrieve structural and physical interaction information from the study. The interaction network can be dynamically filtered based on STRING score to allow differentiation between high and low-confidence interactors. Since OmniPath implements multi-omic databases, we envision that future versions of the app will incorporate additional information from more large-scale interaction databases.

### 2.5 Data reporting

While proteomic data are standardly deposited in conjunction with publication, statistical analyses associated with proteomic studies are often performed with ad hoc workflows or using closed-source applications. QProMS provides a platform for reproducible statistical analysis in proteomics by allowing users to export settings files to be reuploaded in the app for later re-analysis. All settings of statistical tests, as well as names of uploaded files, experimental design and app version are recorded in a human-readable version in the settings file. The analyses can also be exported as a shareable, self-contained html report, which allows users and their collaborators to visualize the data dynamically without accessing the app or installing software, while providing a permanent record of the results. Network visualizations can also be exported for use in Cytoscape. For users who do not wish to upload their results to a web interface, the QProMS pipeline can be run as a ShinyApp on a home server or a local machine by downloading the code provided on GitHub (https://github.com/ieoresearch/QProMS) with a simple command within RStudio. All dependencies are handled by the R package manager, providing the user with a simple installation procedure.

## 3 Conclusions

We have introduced QProMS, a web-based, user-friendly application for quantitative proteomics compatible with a wide range of modern search engine outputs. The app couples state-of-the-art proteomic statistical analysis for both DDA and DIA with functional analysis and network visualization in an online framework that is nevertheless compatible with integration into bioinformatics pipelines. It allows users to simultaneously analyze multiple experimental conditions against each other and easily extract information from the complex, multidimensional proteomics studies generated by modern high-throughput approaches. All results generated by QProMS are shareable, scalable, and reusable, as analyses can be exported as self-contained reports together with machine-readable settings files that capture all parameters and metadata required to reproduce the workflow, in accordance with FAIR principles for research software.

## Supplementary Material

vbag158_Supplementary_Data

## Data Availability

The code for *QProMS* is available at https://github.com/ieoresearch/QProMS. The data and code for the imputation benchmarking can be found at github.com/grandrea/mixed-imputation-benchmark. The app is available at https://shiny.bioserver.ieo.it/app/qproms. The data for the imputation benchmark is available at PRIDE with identifier PXD043638 (Russo *et al.* 2023) and at https://github.com/liangjin0912/proteomics_imputation (Jin *et al.* 2021).
