# Supplementary material for: QproMS: a web application for label-free proteomic data analysis
Source: Bioinform Adv. 2026 Jun 5;6(1):vbag158. doi: 10.1093/bioadv/vbag158 (PMC13278766; doi:10.1093/bioadv/vbag158)
Supplement: vbag158_Supplementary_Data [file vbag158_supplementary_data.zip › QProMS_supplementary_information.docx]

**Supplementary information for QproMS: a web application for label-free proteomic data analysis**

**Benchmarking mixed imputation**

Imputation strategies were evaluated by normalized root mean squared error (NRMSE) between imputed values and ablated values in the original data frame. The dataframe from Jin *et al.* [*(Jin et al., 2021)*](https://paperpile.com/c/9Xkrbm/IXAV) was downloaded from the author’s github as described in the original manuscript (<https://github.com/liangjin0912/proteomics_imputation/blob/master/56%20biological%20replicates_quantitative%20data.xlsx>). The dataset from Russo *et al.* [*(Russo et al., 2023)*](https://paperpile.com/c/9Xkrbm/TKuS) was downloaded from PRIDE using identifier PXD043638. The 2 dataframes are available in supplementary data 1. Protein LFQ intensities were log2-transformed. Data ablation was performed for either dataframe by simulating missing values in DDA. Protein abundances were removed preferentially (75% chance) from the 30% lowest values, however 25% of values were removed fully at random, to simulate stochastic precursor selection in DDA. Data removal was performed 10 times with different random seeds. It was then performed for different missing value ratios (Supplementary figure 1).

For each dataframe, we evaluated 5 imputation strategies: downshifted random distribution imputation (downshift=1.8, scale=0.3); mixed imputation with three different missing at random/missing not at random thresholds (MAR/MNAR threshold); missForest imputation (maxiter= 20, ntree= 100).

In mixed imputation, the MAR/MNAR threshold defines the minimum percentage of valid values present per condition: above this value, the missing value is classified as MAR and imputed a uniform distribution between the maximum and minimum value of the protein in that condition. Below this value, it is imputed with the traditional downshifted random distribution. Results were evaluated based on NRMSE, with normalization applied by the variance of the non-imputed protein abundance values, as defined in the missForest package:


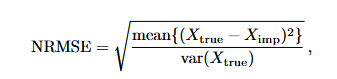


Where X_imp_ is the imputed log2-transformed protein abundance and X_true_ is the original protein abundance.

Mixed imputation consistently outperforms downshifted random distribution imputation at different fractions of missingness.

All code used for benchmarking and generating figure S1 is available at <https://github.com/grandrea/mixed-imputation-benchmark> .


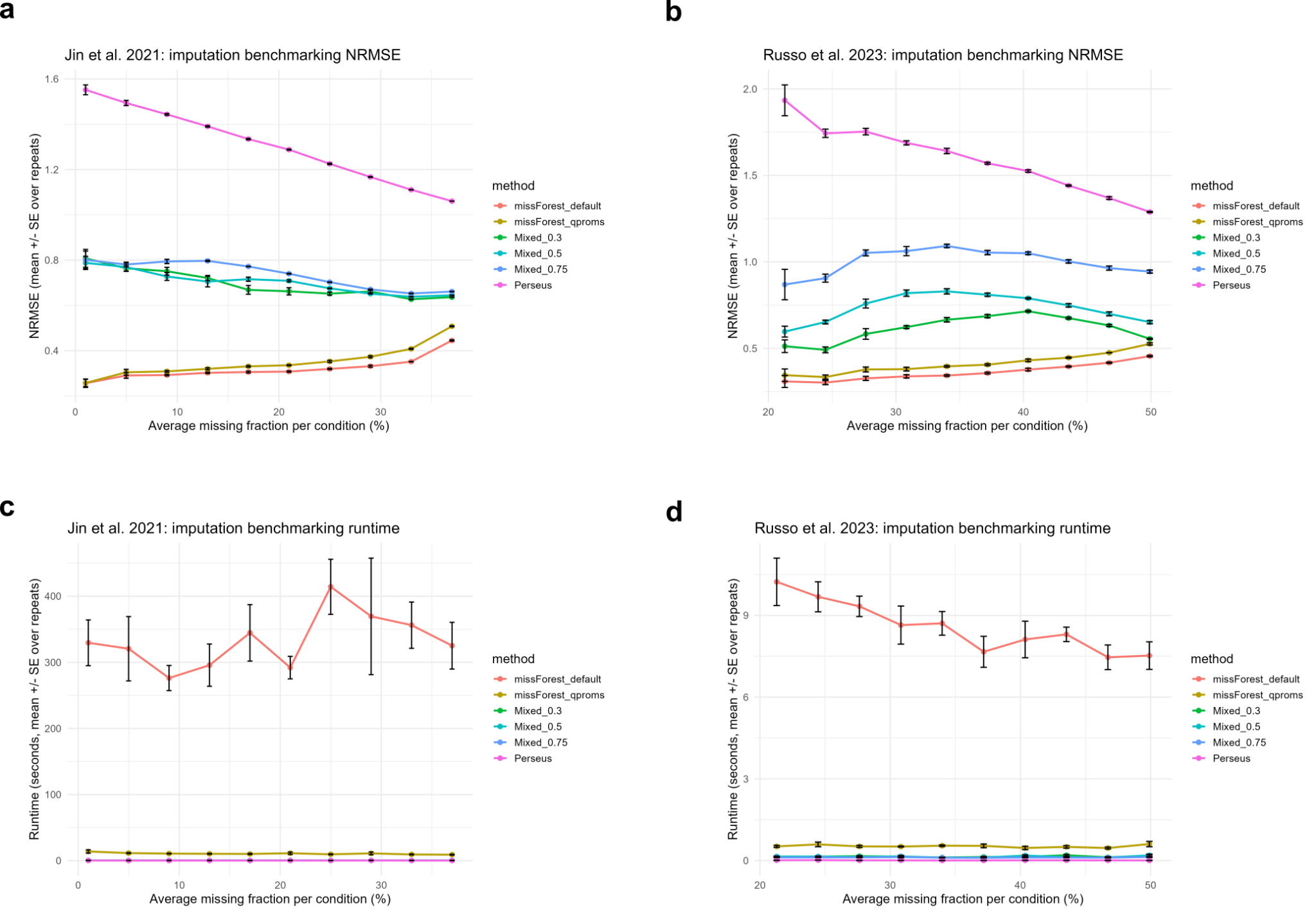


**Supplementary figure S1: benchmarking mixed imputation**

**a)** Evaluation of imputation performance over different fractions of missing data for the 56-biological replicate set published in *Jin et al.* NRMSE=normalized root mean squared error, SE = standard error. N=5. MissForest imputation was performed either with the QProMS settings (maxiter=1, trees=10) or with the full default settings (maxiter=10, trees=100). Mixed imputation was performed with 3 different MAR/MNAR thresholds, 0.3, 0.5 and 0.75. **b)** Evaluation of imputation performance for the dataset of Russo *et al. 2023.* In this case, mixed imputation uses the condition information present in the experimental design and MNAR/MAR is computed separately for each experimental condition. **c)** Runtime evaluation on *Jin et al.* **d)** Runtime evaluation on *Russo et al.*

[Jin,L. *et al.* (2021) A comparative study of evaluating missing value imputation methods in label-free proteomics. *Scientific Reports*, **11**, 1760.](http://paperpile.com/b/9Xkrbm/IXAV)

[Russo,M. *et al.* (2023) Restrictor synergizes with Symplekin and PNUTS to terminate extragenic transcription. *Genes Dev*, **37**, 1017–1040.](http://paperpile.com/b/9Xkrbm/TKuS)
